# Interaction of Poliovirus Capsid Proteins with the Cellular Autophagy Pathway

**DOI:** 10.3390/v13081587

**Published:** 2021-08-11

**Authors:** Anna Zimina, Ekaterina G. Viktorova, Seyedehmahsa Moghimi, Jules Nchoutmboube, George A. Belov

**Affiliations:** 1 Department of Veterinary Medicine, University of Maryland, College Park, MD 20742, USA; azimina@umd.edu (A.Z.); eviktoro@umd.edu (E.G.V.); smahsamoghimi@gmail.com (S.M.); nchoutmboube@gmail.com (J.N.); 2 Virginia Maryland College of Veterinary Medicine, College Park, MD 20742, USA; 3 MIlliporeSigma, 9900 Blackwell Road, Rockville, MD 20850, USA

**Keywords:** enteroviruses, poliovirus, autophagy, LC3 processing, enterovirus replication

## Abstract

The capsid precursor P1 constitutes the N-terminal part of the enterovirus polyprotein. It is processed into VP0, VP3, and VP1 by the viral proteases, and VP0 is cleaved autocatalytically into VP4 and VP2. We observed that poliovirus VP0 is recognized by an antibody against a cellular autophagy protein, LC3A. The LC3A-like epitope overlapped the VP4/VP2 cleavage site. Individually expressed VP0-EGFP and P1 strongly colocalized with a marker of selective autophagy, p62/SQSTM1. To assess the role of capsid proteins in autophagy development we infected different cells with poliovirus or encapsidated polio replicon coding for only the replication proteins. We analyzed the processing of LC3B and p62/SQSTM1, markers of the initiation and completion of the autophagy pathway and investigated the association of the viral antigens with these autophagy proteins in infected cells. We observed cell-type-specific development of autophagy upon infection and found that only the virion signal strongly colocalized with p62/SQSTM1 early in infection. Collectively, our data suggest that activation of autophagy is not required for replication, and that capsid proteins contain determinants targeting them to p62/SQSTM1-dependent sequestration. Such a strategy may control the level of capsid proteins so that viral RNAs are not removed from the replication/translation pool prematurely.

## 1. Introduction

**Figure 1:**
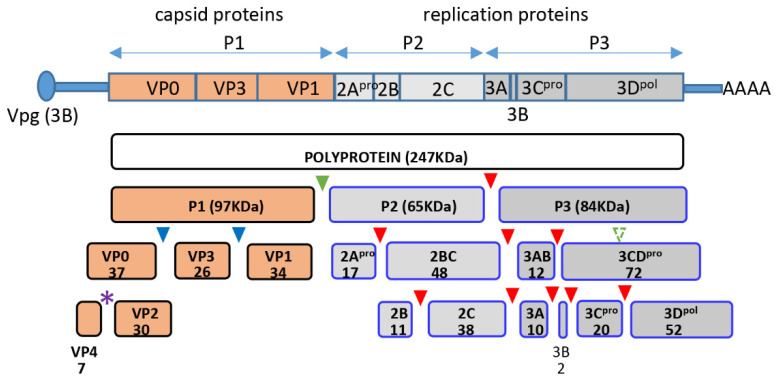
Scheme of poliovirus genome organization and polyprotein processing. Cleavage sites of the protease 2A are marked by green, those of 3C by red, and those of 3CD by blue triangles, respectively. Purple star denotes the autocatalytic cleavage site between VP4 and VP2. Numbers indicate the molecular weight of the corresponding proteins in KDa.

Enteroviruses are ubiquitous human pathogens whose infection may be associated with a variety of pathological conditions, including the development of type I diabetes, temporary or permanent paralysis, fatal encephalitis, and many others. Enteroviruses have non-enveloped virions of icosahedral symmetry containing a single positive-strand RNA genome. The genome RNA codes for one polyprotein which is processed by the viral proteases co- and post-translationally into about a dozen individual peptides. The N-terminal part of the polyprotein contains the precursor of structural proteins P1, which is separated *in cis* from the rest of the polyprotein by the viral protease 2A and is further processed *in trans* by the proteases 3CD and/or 3C into VP0, VP3, and VP1 (Figure 1). Sixty copies of each of these structural proteins form the immature virion, and RNA packaging triggers the final autocatalytic cleavage of VP0 into VP4 and VP2, which stabilizes the virion [1,2,3].

Poliovirus is the best-studied enterovirus, its replication cycle is short, 6–8 h in common cell cultures, making it an excellent model to study the fundamental aspects of enterovirus replication. Poliovirus infection induces profound reorganization of the cellular metabolism, from the inactivation of the nuclear-cytoplasmic trafficking, transcription, and cap-dependent translation, to complete reorganization of the cellular membrane architecture due to rewiring of the lipid and membrane synthesis and trafficking pathways [4,5]. These changes in the infected cell reflect a balance of the processes creating an environment conducive to the viral replication, and those aimed at limiting the viral propagation and communicating the infected status of the cell to the immune system due to activation of the anti-viral mechanisms.

Autophagy is among the cellular membrane metabolism pathways targeted by enteroviruses. Autophagy is a membrane-dependent housekeeping cellular process responsible for the recycling of organelles and metabolites, and it also plays an important role in stress responses, including defense against pathogens. The dynamic adaptation of autophagy to changing cellular environment is mediated by multiple regulatory networks. The hallmark of autophagy is the formation of characteristic double-membrane vesicles (autophagosomes) where the cargo destined for degradation is sequestered. The core autophagy machinery is highly conserved among eukaryotes, but in multicellular organisms, many autophagy-related genes underwent expansion and diversification, and some autophagy-related proteins are specific to multicellular eukaryotes (reviewed in [6,7]). The development of autophagosomes relies on the concerted action of complexes of autophagy proteins. Atg1/ULK kinase complex regulates initiation of autophagosome formation, Atg9 complex coordinates the delivery of lipid material for the autophagosome membrane expansion, Atg14/phosphatidylinositol 3-kinase (PI3K) complex mediates autophagosome nucleation and its activity is required for the recruitment of multiple PI3P-binding proteins. Finally, closely interconnected Atg12 and Atg8 ubiquitin-like conjugation systems coordinate autophagosome development and cargo recruitment (reviewed in [8,9,10]).

Human cells express six members of the Atg8 family proteins which are subdivided into subfamilies based on their structural homology. The LC3 subfamily of Atg8 proteins includes LC3A, LC3B, and LC3C. These proteins are expressed ubiquitously, although LC3C is preferentially expressed in the lungs [11,12,13]. All Atg8 family proteins have a central conserved ubiquitin-like domain that shares with ubiquitin the 3D structure but not the primary sequence, and a less conserved N-terminal part which to the large part determines the specific interactions of different Atg8-like proteins [14,15,16,17,18]. LC3 proteins are expressed as precursors that undergo cleavage by Atg4 proteases to expose the C-terminal glycine generating LC3I form. The LC3I serves as a substrate for a ubiquitin ligase-like complex that attaches it to the amino group of phosphatidylethanolamine generating the lipidated LC3II form decorating the membrane surface. The ratio between the LC3I and the LC3II forms is often used to monitor the activation of autophagy [19]. The lipidated LC3II proteins exposed on the growing autophagosome membrane mediate cargo recruitment through interaction with selective autophagy receptors (SAR). More than 30 SARs are expressed in mammalian cells, some of them like p62/SQSTM1 are implicated in the recruitment of diverse types of cargo to autophagosomes, from proteins to bacteria, while others are more selective. SARs have an LC3-interacting region (LIR) and also contain other protein–protein interaction domains including those that mediate oligomerization and interaction with ubiquitinated substrates. SARs recognize ubiquitin and other signatures on the cargo destined for autophagosomal degradation and coordinate the cargo delivery to growing autophagosomes through the interaction of LIR with Atg8 proteins (reviewed in [20]). The final step in the autophagy pathway is the fusion of autophagosomes with lysosomes where the cargo is exposed to the degradation machinery. SARs are degraded with the autophagosomal content so that changes in the concentration of proteins like p62/SQSTM1 are used to monitor the so-called autophagic flux, i.e., the completion of the degradative pathway [19].

While the manipulation of the autophagy pathway by enteroviruses has been studied extensively ever since it was discovered in early 2000 [21,22,23], the data on the mechanistic contribution of autophagy to the development of enterovirus infection remain controversial. In different enterovirus/cell type systems, autophagy has been reported to either directly support the virus replication, or to modulate the anti-viral signaling thus either suppressing or promoting the replication [24,25,26,27,28,29]. However, the consensus picture suggests that enterovirus replication may be accompanied by the activation of the early steps of the autophagy cascade, often bypassing conventional signaling checkpoints [30,31], and by the inhibition of the final fusion of maturing autophagosomes with lysosomes [22,23,32,33]. It was recently established that sequestration of the progeny enterovirus virions in the autophagosome-like vesicles that have escaped fusion with lysosomes promotes their maturation and a non-lytic release from infected cells [34,35,36,37]. This scenario suggests that enterovirus capsid proteins may have evolved specific features enabling interaction with the elements of the cellular autophagy machinery to promote nascent virions recruitment into autophagosome-like structures that support the final steps of the virion maturation and dissemination.

In this work, we followed an intriguing serendipitous observation of a strong cross-reactivity of a monoclonal antibody against a cellular protein LC3A with a higher molecular weight protein appearing in HeLa cells infected with poliovirus. We identified this cross-reacting protein as the capsid protein VP0 and localized the LC3A-like epitope at the VP4-VP2 cleavage site. This observation prompted us to further investigate the role of capsid proteins in the development of autophagy during infection. Taking advantage of our recently developed replicon packaging system [38], we examined the induction of autophagy in cells infected with poliovirus or with a packaged replicon RNA coding only for the non-structural proteins (P2P3). We observed a highly cell-type-specific pattern of autophagy development upon either replicon or virus infection. The viral replication was not affected by the level of autophagy activation and no significant association of the poliovirus replication proteins or dsRNA signal with either LC3B or p62/SQSTM1 was detected in HeLa cells. Moreover, the level of LC3B clustering indicative of the activation of autophagy decreased as infection progressed, and inversely correlated with the amount of the viral antigens in individual cells, indicating that infection blocks rather than activates autophagy and/or that the elevated level of autophagy is detrimental for the establishment of infection. Surprisingly, the replicon replication in HEK293 cells but not in other cell cultures tested was severely compromised compared to the full-length poliovirus RNA, suggesting that expression of capsid proteins likely modulates cellular processes that may be limiting for replication in specific cell types. Individually expressed VP0 and an uncleaved capsid protein precursor, P1, were recruited to p62/SQSTM1-positive structures but did not undergo a significant degradation through either autophagy or proteasome-dependent pathways. Additionally, in infected cells, we detected a strong colocalization of the virion signal with p62/SQSTM1 at the beginning of virion accumulation that was lost as the infection progressed. Together, our data suggest that activation of autophagy is not directly required to support the viral replication, and that capsid proteins contain determinants mediating their sequestration in a p62/SQSTM1-dependent manner. Such sequestration would provide enteroviruses with a mechanism of regulating the amount of accessible capsid proteins, preventing premature encapsidation of the replicating RNA early in infection.

## 2. Materials and Methods

### 2.1. Cells, Poliovirus and Polio Replicon

HeLa, and HEK293 cells were grown in a high-glucose DMEM supplemented with pyruvate, non-essential amino-acids and 10% fetal bovine serum (FBS). A549 cells were grown in F-12K medium supplemented with 10% FBS. HeLa cells were from Dr. Ellie Ehrenfeld (NIH), HEK293 and A549 cells were from ATCC (Mannassas, VA, USA). Poliovirus type I Mahoney was propagated in HeLa cells and the titer was determined by plaque assay. P2P3 polio replicon construct is the same as type I Mahoney poliovirus, but the coding sequence for the capsid protein precursor P1 was removed. The construct was made using the plasmid pXpA-SH containing the full-length cDNA of poliovirus type I Mahoney described previously [39,40]. Replicon construct packaging and propagation were performed essentially as described in [38]. Briefly, in the first round, the replicon RNA was transfected into HeLa cells previously infected with a Newcastle Disease virus (NDV) vector expressing poliovirus capsid protein precursor P1 and the protease 3CD [41]. In subsequent amplification rounds, the packaged polio replicon was used to infect the cells previously infected with the NDV expressing polio capsid proteins. Importantly, NDV infection in HeLa cells does not produce infectious progeny, and all the replicon preparations were tested in a plaque assay to confirm the absence of infectious poliovirus. The titer of the infectious replicon was determined by serial dilutions, similar to a plaque assay, and identification of the number of individually infected cells by immunofluorescence. Work with all viruses was performed upon permission of and in strict adherence to the rules established by the University of Maryland Institutional Biosafety Committee.

### 2.2. Plasmids

Plasmids pVP0-EGFP and pVP012A-EGFP coding for wt VP0 or VP0 with alanines substituting the amino-acids at the VP4/VP2 autocatalytic site, respectively, were made using pEGFP-N1 vector (Clontech, Mountain View, CA, USA) and the corresponding constructs coding for poliovirus type I Mahoney VP0 with a codon sequence optimized for translation in human cells synthesized by GeneArt (Invitrogen, Waltham, MA, USA). Plasmid pM1-P1 was made using a pM1-MT vector for a high level of mammalian expression (Roche, Basel, Switzerland) and a PCR-amplified P1 sequence of poliovirus type I Mahoney from pXpA-SH [39,40]. Plasmids pM1-P1-12A and pXpA-SH-P1-12A containing P1 with 12A mutation were made by cloning a DNA fragment containing 12A mutation synthesized by GeneArt (Invitrogen, Waltham, MA, USA) into pM1-P1 or pXpA-SH, respectively. Cloning details are available upon request. Poliovirus or replicon RNAs were synthesized using T7 Megascript transcription kit (Ambion, Austin, TX, USA), purified and transfected into HeLa cells using Mirus mRNA transfection reagent (Mirus Bio, Madison, WI, USA) essentially as described in [38].

### 2.3. Antibodies

Mouse monoclonal antibodies against poliovirus antigens VP3, VP1, 2B, 2C, and 3A, and rabbit polyclonal antibodies against 3B were a gift from Prof. Kurt Bienz, University of Basel, Switzerland, and have been partially described in [42,43,44]. Rabbit polyclonal antibodies against poliovirus 3D were described in [45]. Rabbit monoclonal antibodies against LC3A, LC3A/B, and mouse monoclonal antibodies against LC3B were from Cell Signaling. Mouse monoclonal antibody and rabbit polyclonal antibodies against p62/SQSTM1 were from Santa Cruz Biotechnology and Sigma-Aldrich, respectively. Mouse monoclonal antibody against Ubiquitin was from BioLegend, San Diego, CA, USA. For immunostaining, rabbit monoclonal antibody against LC3A/B and mouse monoclonal antibody against LC3B, or rabbit polyclonal or mouse monoclonal anti-p62/SQSTM antibodies were used together with the anti-viral mouse or rabbit antibodies, respectively. Mouse monoclonal antibody J2 against dsRNA was from English and Scientific Consulting. Humanized chimpanzee monoclonal antibody A12 that recognizes the assembled polio virions [46] was generously provided by Dr. Konstantin Chumakov (FDA). Rabbit polyclonal anti-GFP antibodies were from Abcam. Secondary antibody conjugates with Alexa dyes were from Molecular Probes. Anti-β-actin antibody conjugated to horseradish peroxidase (HRP) was from Sigma-Aldrich. Secondary antibody conjugates with HRP were from Cell Signaling (anti-rabbit) or KPL (anti-mouse).

### 2.4. Western Blotting

Cells were lysed either in a Tris-HCl buffer with 0.5% Triton-X100 (for assessment of the activation of autophagy), or in a standard RIPA buffer (for assessment of the effect of autophagy and proteasome inhibition on P1 accumulation) supplemented with a protease inhibitor cocktail (Sigma-Aldrich, St. Louis, MO, USA). In the former case, the lysate was clarified by centrifugation, in the latter case the lysate was sonicated to break down nuclear DNA. The proteins were denatured and resolved on either 12% Tris-glycine SDS or 4–15% Tris-glycine gradient gels (Bio-Rad, Hercules, CA, USA), transferred to a PVDF membrane, and analyzed with the indicated antibodies. Digital images of Western blots developed with ECL Select luminescent substrate (GE Healthcare, Chicago, IL, USA) were obtained with a C500 imager (Azure Biosystems, Dublin, CA, USA). Quantitative analysis of Western blots was performed by Image Studio software (Li-cor, Lincoln, NE, USA).

### 2.5. Co-Immunoprecipitation

HeLa cells were transfected with a plasmid pVP0-EGFP or an EGFP-expressing vector and grown on 35-mm Petri dishes overnight. The next day a pull-down assay was performed using anti-GFP nanobody beads (Chromotec, Munich, Germany) according to the manufacturer’s protocol. Western blot was developed with anti-p62/SQSTM1 antibodies to assess the VP0- p62/SQSTM1 interaction and then with anti-EGFP antibodies to assess the efficiency of the pull-down.

### 2.6. Microscopy

HeLa cells grown on coverslips were fixed with 4% formaldehyde in phosphate-buffered saline (PBS) for 20 min and washed three times with PBS. Staining was performed after permeabilization with 0.2% Triton-X100 in PBS. Primary and secondary antibodies were diluted in PBS supplemented with 3% of membrane blocking reagent (Amersham, England). Confocal images were taken with a Zeiss LSM 510 microscope. For quantitative cell phenotype analysis at least 50 cells were analyzed from randomly chosen fields.

### 2.7. Data Analysis

Quantitative data were analyzed using unpaired t-test in the GraphPad Prism software. Graphs show average value and standard deviation. The difference between the sets of data was considered statistically significant at *p* values < 0.05.

### 2.8. Digital Image Processing

Digital microscopy images were converted into TIFF format using Zeiss Zen software. Digital Western blot and microscopy images were processed in Adobe Photoshop software; all changes were applied to the whole image.

## 3. Results

### 3.1. Poliovirus Capsid Protein VP0 Contains an LC3A-like Epitope

**Figure 2:**
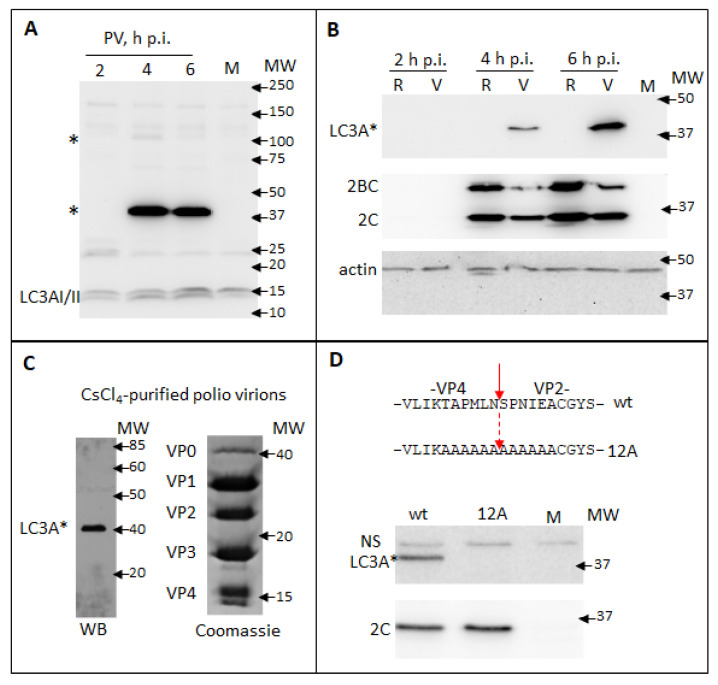
Poliovirus capsid protein VP0 contains an LC3A-like epitope. (**A**) HeLa cells were infected with poliovirus at an MOI of 10 and the samples were collected at the indicated times post-infection. Mock-infected sample is collected at 6 h. Western blot was developed with a rabbit monoclonal anti-LC3A antibody (Cell Signaling). LC3A * designates abnormal higher molecular weight LC3A signals. (**B**) HeLa cells were transfected with either full-length poliovirus RNA (V) or replicon RNA (R) coding for only P2P3 replication proteins and developed with the same anti-LC3A antibody as in A (upper panel). Staining with anti-polio 2C antibody shows polio replication (middle panel). Actin is shown as a loading control (lower panel). (**C**) Proteins from poliovirus virions purified through CsCl_4_ gradient were resolved on an SDS-PAGE gel and either subjected to a Western blotting with the anti-LC3A antibody (left), or visualized with a Coomassie stain (right). (**D**) Top—amino-acid sequences of the VP4/VP2 cleavage site in VP0 and the alanine substitutions in the 12A mutant. HeLa cells were transfected with either wt polio RNA or the RNA with 12A substitution and the cell lysates were subject to Western blots with either anti-LC3A (top panel) or anti-polio 2C antibodies (bottom panel).

While assessing in a Western blot the processing of a cellular autophagy protein LC3A upon infection of HeLa cells with poliovirus, we observed infection-specific LC3A signals at ~100 KDa and ~40 KDa, which is significantly higher than the endogenous LC3AI or LC3AII (14–17 KDa). The ~100 KDa signal was of variable intensity in different experiments, while the ~40 KDa signal was always much stronger than the signal for *bona fide* LC3A (Figure 2A and data not shown). This pattern is strongly indicative that a viral protein of ~40 KDa contains an LC3A-like antigen and the transient nature of a ~100 KDa signal corresponds to the processing of a larger piece of a viral polyprotein containing this epitope (see Figure 1). To discriminate if the signal is associated with structural or replication proteins, we transfected cells with either full-length poliovirus RNA or with a replicon RNA coding for only P2P3 proteins. The latter RNA is fully replication-competent, but obviously, its translation does not produce capsid proteins. As can be seen from Figure 2B, the high molecular weight LC3A signal was observed only upon replication of the full-length poliovirus genome, even though the level of expression of other viral proteins was higher in the replicon sample. The only capsid protein fragment corresponding to ~40 KDa is VP0, an uncleaved precursor of VP4 and VP2 (see Figure 1). We analyzed capsid proteins from poliovirus virions purified through the CsCl gradient. The Western blot with anti-LC3A antibodies again showed a strong signal only at ~40 KDa. The Coomassie staining of a gel with resolved capsid proteins demonstrated a distinct VP0 band at ~40 KDa, in accordance with the previous reports that virions contain some unprocessed VP0 molecules [47,48] (Figure 2C). Since neither VP2 nor VP4 was recognized by the anti-LC3A antibody, we concluded that the epitope is overlapping the junction between the two proteins. To confirm the location of the LC3A-like epitope we generated a mutant polio RNA with alanine substitutions spanning the VP4/VP2 cleavage site on each side (12A mutant, Figure 2D). As expected, upon 12A RNA transfection no infectious virus was recovered (data not shown), but the mutant RNA was fully replication competent. Analysis of the lysates of HeLa cells transfected with the wt and the mutant RNAs showed that both RNAs replicated similarly, as evidenced by the accumulation of a viral protein 2C, but the high molecular weight LC3A-specific signal was present only in the wt sample (Figure 2D).

Thus, an LC3A-like epitope spans the VP4/VP2 cleavage site in the poliovirus capsid protein VP0. This result also highlights the importance of validation of possible cross-reactivity of antibodies.

### 3.2. Individually Expressed VP0 and P1 Are Recruited to p62/SQSTM1-Positive Structures

**Figure 3:**
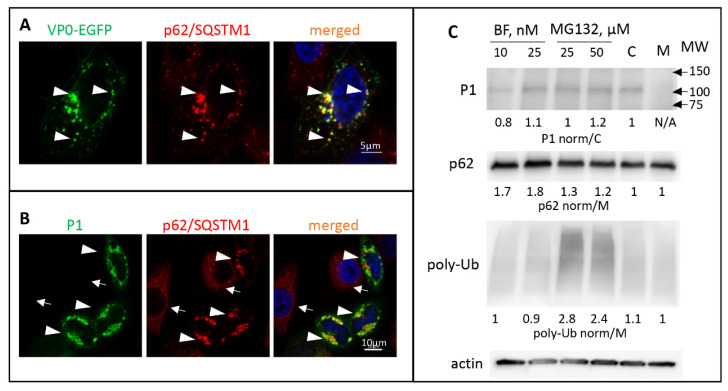
Poliovirus capsid proteins are sequestered in a p62/SQSTM-dependent manner. (**A**) HeLa cells expressing VP0-EGFP fusion (green) stained with an anti- p62/SQSTM antibody (red) Arrowheads show colocalization of both signals. Nuclear DNA is stained with Hoechst 33,342 (blue). (**B**) HeLa cells expressing the whole capsid protein precursor P1 stained with anti-polio VP1 (green) and anti- p62/SQSTM (red) antibodies. Arrowheads and arrows show cells that either express or do not express P1, respectively. Note the concentration of p62/SQSTM signal in the former compared to the mostly diffuse staining in the latter. (**C**) HeLa cells were transfected with a plasmid expressing the whole P1 capsid protein precursor, and at 18 h post-transfection an inhibitor of lysosome acidification bafilomycin or a proteasome inhibitor MG132 were added at the indicated concentrations. C-control cells not treated with inhibitors. Total cell lysate was prepared after six hours of incubation with the inhibitors and analyzed with the anti-VP3 antibodies in a Western blot to assess the accumulation of P1 and with anti-p62 and anti-ubiquitin antibodies to confirm the efficacy of the inhibitor treatment. Quantitation shows P1 signal normalized to that in the sample not treated with inhibitors (**C**), p62 and polyubiquitin signals are normalized to those in the sample transfected with an empty vector (M). Actin is shown as a loading control.

No obvious amino-acid sequence resemblance of LC3A to the VP4/VP2 cleavage site could be detected; nevertheless, the cross-reactivity of VP0 with a monoclonal anti-LC3A antibody indicates a region of local structural similarity of the two proteins. To assess the interaction of VP0 with the elements of the cellular autophagy machinery we generated a construct coding for poliovirus VP0 C-terminally fused to EGFP. Such fusion arrangement recapitulates the most N-terminal position of VP0 in poliovirus polyprotein. The cells with a low level of VP0-EGFP expression demonstrated diffuse cytoplasmic staining, while in cells with a higher level of expression the signal was concentrated in distinct punctae of heterogeneous size. These VP0-EGFP punctae were extensively colocalized with endogenous p62/SQSTM1 (Figure 3A, arrowheads). To see if VP0 can directly interact with p62/SQSTM1, we expressed the VP0-EGFP fusions and performed a pull-down assay with beads conjugated to an anti-GFP nanobody. No p62/SQSTM1 signal was detected in the pull-down material, ruling out a strong direct interaction of the two proteins (data not shown).

The precursor of all enterovirus capsid proteins P1 is separated from the rest of the polyprotein *in cis* by the protease 2A, and, at least before the substantial accumulation of proteases 3C and/or 3CD that can process it, P1 should exist as a single polypeptide (see Figure 1). We expressed poliovirus P1 fragment individually and assessed its cellular targeting. Like with the expression of VP0-EGFP fusion, we observed a significant accumulation of P1 in p62/SQSTM1-positive structures (Figure 3B, arrowheads. Please note that in cells not expressing P1 the distribution of p62/SQSTM1 is much more diffuse, arrows). Surprisingly, the cellular distribution and the colocalization with p62/SQSTM1 of the VP0(12A)-EGFP or P1(12A) with the alanine substitutions spanning the VP4/VP2 cleavage site was not noticeably different from that of the wt proteins (data not shown). We did not observe any significant effect of either an autophagy inhibitor bafilomycin or a proteasome inhibitor MG132 on the accumulation of P1 in transfected cells, suggesting that P1 sequestered in p62/SQSTM1-positive structures does not rapidly enter the degradative pathways (Figure 3C). Collectively, these data suggest that VP0 contains multiple determinants targeting the capsid protein precursor P1 for sequestration in a p62/SQSTM1-dependent manner.

### 3.3. The Development of Autophagy upon Polio Infection Is Cell Type Specific

**Figure 4:**
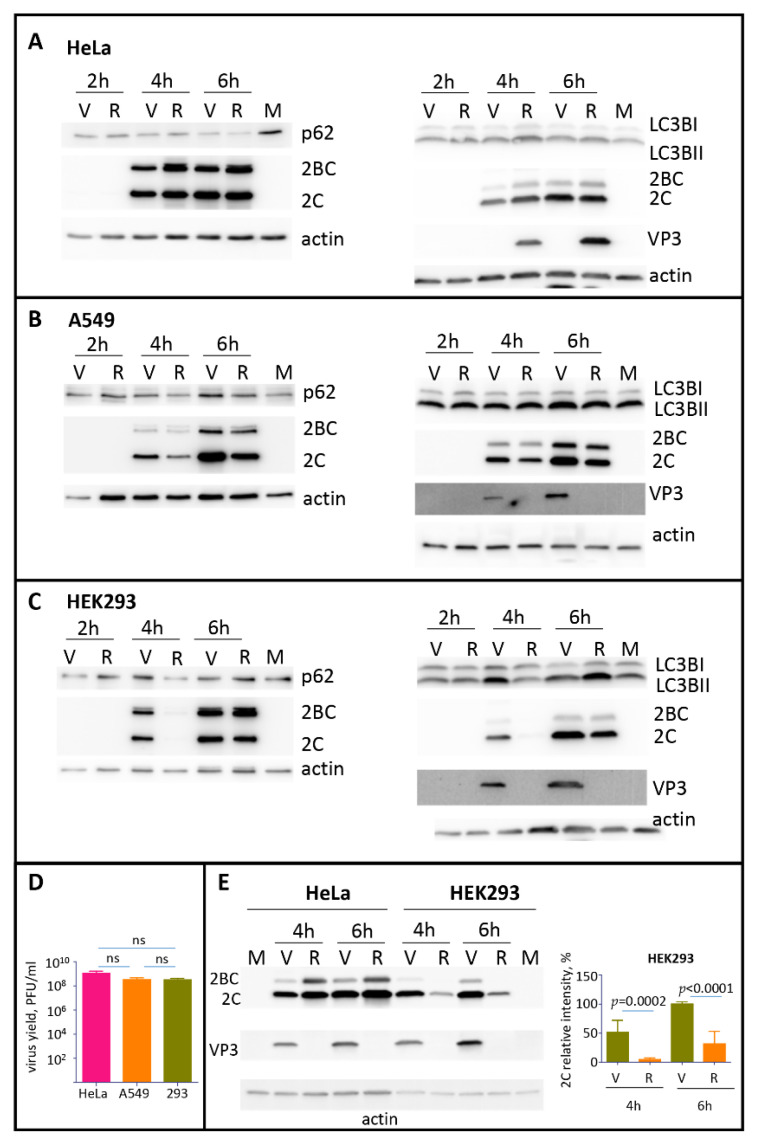
The development of autophagy upon polio infection is cell type specific. (**A**–**C**) HeLa, A549, or HEK293 cells, respectively, were infected with the MOI of 25 of either poliovirus (V) or encapsidated P2P3 replicon (R), and the cells were lysed at the indicated times post-infection. Lysates from mock-infected cells were prepared at 6 h. The same lysates were resolved either on 12% (left panels) or on 4–15% gradient gels (right panels) and analyzed in Western blots with the indicated antibodies. Staining with anti-2C antibody shows polio replication, staining with anti-VP3 antibody confirms the absence of capsid protein expression in cells infected with the replicon construct, actin is shown as a loading control. (**D**) HeLa, A549 and HEK 293 cells were infected with 25PFU of poliovirus and the virus yield at 6 h p.i. was determined by plaque assays. The difference in the titers between any of the samples was non-significant (ns). (**E**) HEK293 and HeLa cells were infected in parallel with the same preparations of either poliovirus (V) or encapsidated P2P3 replicon (R), collected at the indicated times post-infection and analyzed in a Western blot with anti-polio 2C and VP3 (capsid protein) antibodies. Actin is shown as a loading control. Note a significantly compromised replication of replicon compared to poliovirus in HEK293 but not HeLa cells. Quantitation shows 2C signals normalized to that in the virus-infected sample at 6 h p.i. from three independent experiments of infection of HEK293 cells with encapsidated replicon and poliovirus.

The formation of p62/SQSTM1 clusters with VP0-EGFP or P1 suggests that capsid proteins can contribute to the modulation of the autophagy pathway during the infection. To assess the development of autophagy in the absence and in the presence of expression of capsid proteins, we infected different cell lines with poliovirus, or encapsidated replicon RNA coding for only the P2P3 replication proteins. The infection was performed with the same multiplicity of infection (MOI) of 25 of plaque-forming units, or infectious units of poliovirus or encapsidated replicon, respectively. We monitored by Western blot the processing of LC3B and the degradation of p62/SQSTM1 proteins as the signals for the initial and the late stage of the degradative autophagy pathway, respectively. In HeLa cells (human cervical carcinoma), we observed a significant reduction of the p62/SQSTM1 signal as early as 2 h p.i., which was further disappearing towards the end of infection (6 h p.i.), as well as a slight increase of the LC3BII form in all infected samples. No significant differences were detected between the samples infected with poliovirus or P2P3 replicon (Figure 4A). In A549 cells (human lung carcinoma), neither poliovirus nor replicon infection noticeably affected the amount of p62/SQSTM1 and the processing of LC3B (Figure 4B). In HEK293 cells (human embryonal kidney) p62/SQSTM1 was similarly stable during the time course of infection. The amount of the LC3BII form increased in poliovirus-infected sample at 4 h p.i. and in both, poliovirus and replicon-infected samples at 6 h p.i. (Figure 4C). At the same time, despite the different patterns of LC3B processing and p62/SQSTM1 degradation, all these cell cultures generated similar yield of poliovirus progeny by 6 h p.i. (Figure 4D). Interestingly, we repeatedly observed a significantly lower replication of the replicon construct compared to the full-length polio RNA in HEK293 cells but not in other cell types, especially at the early times post-infection (Figure 4C, compare the signal for a polio antigen 2C in replicon and virus samples at 4 h p.i.). To confirm this phenomenon, we infected in parallel HeLa and HEK293 cells with the same preparations of poliovirus and encapsidated replicon. As evidenced from Figure 4E, the replicon replication was indeed significantly compromised in HEK293 but not in HeLa cells.

Thus, the autophagy machinery responds differently to poliovirus infection in different cell types, and the expression of capsid proteins can be important for the establishment of replication in specific cell types.

### 3.4. Investigation of the Association of Polio Replication Complexes and Virions with LC3B or p62/SQSTM Signals in Infected Cells

**Figure 5:**
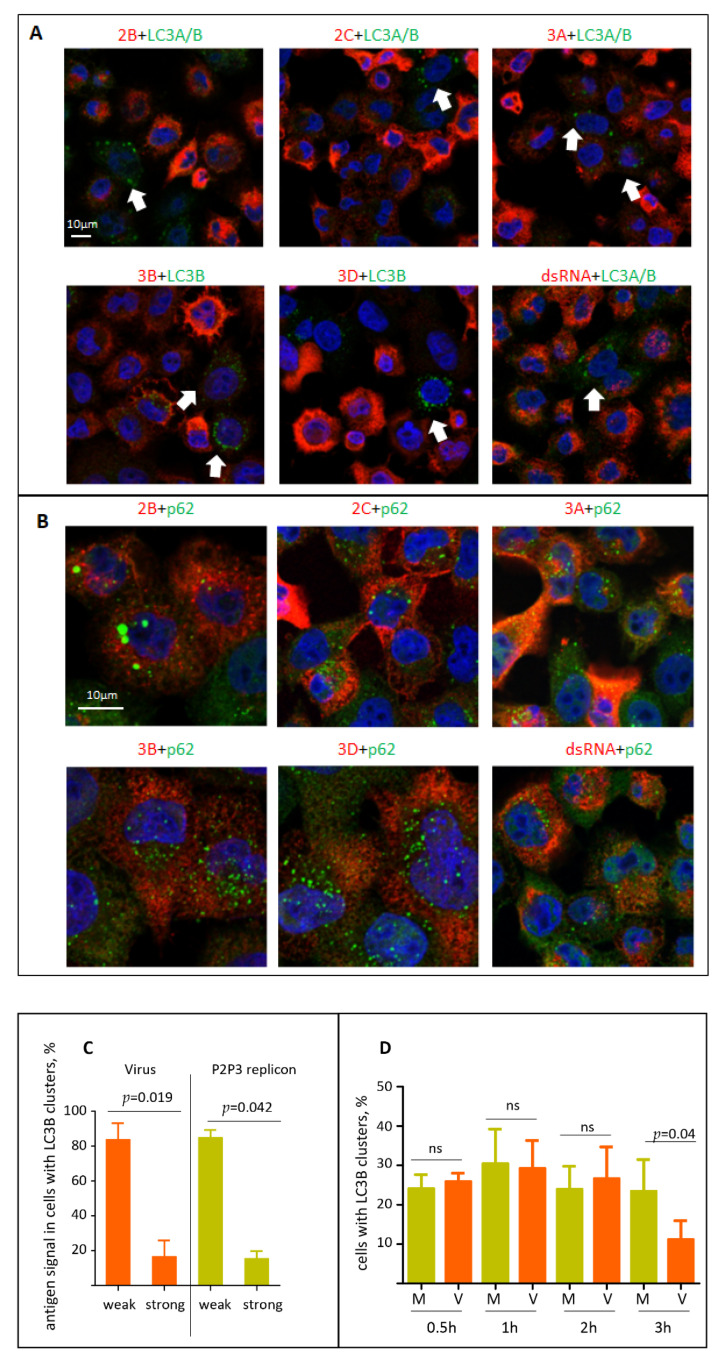

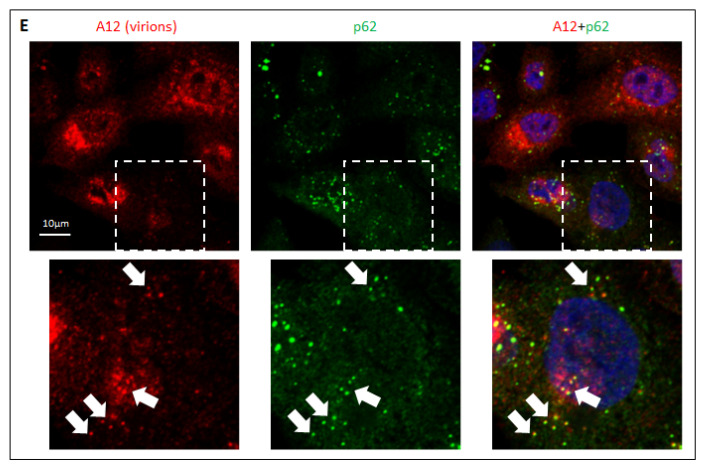
Localization of the viral antigens and autophagy proteins in infected cells. (**A**) HeLa cells were infected with an MOI of 25 of encapsidated P2P3 replicon, fixed at 4 h p.i. and processed for immunofluorescence with either rabbit monoclonal anti-LC3A/B or mouse monoclonal anti-LC3B (green) antibodies and the indicated polio antigens (red). Nuclear DNA is stained with Hoechst 33,342 (blue). (**B**) Same as in A but with either rabbit polyclonal or mouse monoclonal anti- p62/SQSTM (green) antibodies. (**C**) HeLa cells were infected with an MOI of 25 of either encapsidated P2P3 replicon or poliovirus, and at 4 h p.i. stained with antibodies against polio antigens 3D or 3B (rabbit polyclonal), and LC3B (mouse monoclonal). The level of viral antigen expression was quantified in at least 50 cells with LC3B clusters from multiple random fields of view. (**D**) HeLa cells were infected (V) or mock-infected (M) with an MOI of 25 of poliovirus, and at the indicated time post infection stained for LC3B. The percentage of cells with LC3B clusters was quantified from at least 100 cells per sample from multiple random fields of view. (**E**) HeLa cells were infected with the MOI of 25 of poliovirus, fixed at 4 h p.i. and processed for immunofluorescence with a monoclonal antibody A12 (red) recognizing assembled polio virions or virus-like particles and an antibody against p62/SQSTM (green). Nuclear DNA is stained with Hoechst 33,342 (blue). Arrows show colocalization of A12 and p62/SQSTM signals in cells at the early stages of virion accumulation (the selected area is shown).

The current data on whether the activation of autophagy is required to support the functioning of the enterovirus replication complexes remain controversial. We first analyzed if the viral replication proteins or dsRNA signal may be associated with LC3B or p62/SQSTM signals in cells infected with the P2P3 replicon, i.e., in the system where active replication is established but the virion production is absent. The available panel of anti-poliovirus antibodies includes those that recognize 2B, 2C, 3B, 3A and 3D, thus covering all the final and intermediate products of the processing of the P2P3 polyprotein fragment, except for proteases 2A and 3C (see Figure 1). None of the viral replication antigens tested (2B, 2C, 3A, 3B, 3D, dsRNA) noticeably colocalized with either LC3A/B, LC3B, or p62/SQSTM signals (Figure 5A,B). On the contrary, the cells with LC3 clustering were those with a significantly lower level of the viral antigens compared to those without LC3 punctae (Figure 5A, arrows). Quantitation of the level of two viral antigens, 3D and 3B, in cells with LC3B clustering confirmed that in both virus- and replicon-infected samples most of cells with LC3B clusters had low to non-detectable levels of the viral antigens at 4 h p.i. (Figure 5C). We further quantified the dynamics of the cells with LC3B clustering during the early stages of poliovirus infection. Up to 2 h p.i. there was no difference between mock-infected cells and those infected with an MOI of 25 of poliovirus, with 25–30% of cells showing LC3B punctae. At 3 h p.i. in the virus-infected sample the number of cells with LC3B clusters dropped significantly (Figure 5D). We conclude that activation of autophagy is unlikely to be required to directly support the viral replication, on the contrary, the high level of autophagy activation seems to be detrimental to the establishment of infection.

Finally, we analyzed the localization of polio virions using a hybrid human–chimpanzee monoclonal antibody A12 that recognizes a conformational epitope present only in fully assembled poliovirus virions or virus-like particles [46]. As with the replication antigens, the signal for virions inversely correlated with the LC3B clustering, and in the few cells where both signals could be detected no colocalization between the two antigens existed (data not shown). Yet, we observed a strong colocalization of A12 and p62/SQSTM signals, but only in those cells where the A12 signal was relatively weak, i.e., cells where the accumulation of the virions is at the early stage (Figure 5E, inset, arrows). Such colocalization was lost in cells with a strong A12 signal (Figure 5E).

Collectively, our data suggest that autophagy is unlikely to be required to directly support the viral replication, but, rather, the development of infection suppresses the activation of autophagy. They also indicate that the capsid proteins contain determinants targeting them to sequestration in a p62/SQSTM-dependent manner, whether as capsid precursors or assembled capsids, but such sequestration is overcome upon the accumulation of the capsid proteins at the later stages of infection.

## 4. Discussion

Viruses are ultimate parasites and have to actively manipulate the cellular metabolism to support the replication and to counter the anti-viral responses. This implies that the viral proteins have to contain specific elements allowing them to engage in interactions with the cellular factors required to reorganize the cellular metabolism and create an environment favorable for the development of infection. Here we identified a region in the poliovirus structural protein VP0 which was specifically recognized by a commercial monoclonal antibody against a cellular protein LC3A, an Atg8-like protein whose best-studied function is engagement in the early steps of the autophagosome formation [13,49]. The LC3A-like epitope overlaps the autocatalytic cleavage site between the VP4 and VP2. Unexpectedly, no substantial amino-acid similarity could be detected between VP0 and LC3A sequences. Still, similar 3D structures in proteins with different primary amino-acid sequences is a well-known phenomenon. In particular, the Atg8 proteins, including LC3A, contain a ubiquitin-like structural domain without a significant sequence homology among those proteins and ubiquitin [17].

Since VP0 was recognized by the anti-LC3A antibody in a standard Western blot after SDS-PAGE, i.e., after harsh denaturing treatment, the local epitope structure must be very stable. Although we did not find any specific function of this epitope in our experiments, it is tempting to speculate that it may be engaged in the interaction with cellular proteins. VP4 is the most N-terminal sequence of the viral polyprotein and is only 69 amino-acids long in poliovirus; thus, the LC3A-like epitope must emerge from the translating ribosomes before the whole capsid protein precursor P1 (881 amino-acids) is synthesized and can be cleaved in cis from the growing polyprotein by the protease 2A. Interaction of the N-terminal part of the polyprotein containing the LC3A-like epitope with some cellular factors may mediate specific subcellular targeting of the complex of the viral RNA with translating ribosomes. Supporting the possible importance of capsid protein-mediated interactions with cellular factors is our observation of a significantly compromised establishment of replication of the replicon construct lacking the capsid protein-coding region compared to the full-length poliovirus RNA in HEK293 cells. The mechanism underlying this phenomenon warrants further investigation.

Individual expression of VP0 and P1 induced a significant change in the cellular distribution of p62/SQSTM, a protein mediating recruitment of ubiquitinated cargo into autophagosomes [20]. The p62/SQSTM signal in cells expressing those viral proteins changed from mostly diffuse cytoplasmic staining with small foci into large punctae, and these punctae were extensively colocalized with the viral capsid proteins. The mechanism of formation of these p62/SQSTM-positive structures containing viral capsid proteins remains to be established, but it is unlikely that such sequestration of the viral proteins is associated with their active targeting for degradation, as neither an inhibitor of autophagy bafilomycin nor a proteasome inhibitor MG132 affected the level of P1 accumulation in the cells.

Previously, it was established that individually expressed poliovirus proteins 2BC and 3A trigger the autophagy pathway, and at least partially colocalize with autophagosomal and endosomal markers [21,22]. Colocalization of a structural protein VP1 and a replication protein 2C of enterovirus A71 as well as VP1, 2B, 2C and 3A of foot and mouth disease virus, a distantly related picornavirus from the Aphthovirus genus, with the LC3 signal have also been reported [50,51,52]. These data suggest that the elements of cellular autophagy may be directly involved in supporting the functioning of the replication complexes, or progeny RNA encapsidation. Here we investigated the development of autophagy in different cell lines infected with either poliovirus or an encapsidated polio replicon RNA coding for only the replication proteins of the P2P3 polyprotein fragment. We observed a highly cell-type-specific pattern of processing of LC3B and degradation of p62/SQSTM, the markers of autophagy initiation and completion, respectively. Despite the significant differences in the development of autophagy, all the cell cultures supported a similar level of poliovirus replication. Moreover, we did not observe any noticeable colocalization of the signals of dsRNA, or poliovirus replication antigens 2B, 2C, 3B, 3A and 3D, which cover all the P2P3 processing fragments except proteases 2A and 3C, with either LC3B or p62/SQSTM. Our data strongly suggest that the development of autophagy does not directly contribute to the formation or functioning of the viral replication complexes but rather represents a cell type-specific response to infection. Indeed, the cell-type-specific role of autophagy in enterovirus infection has been reported. Atg7-dependent activation of autophagy in neuronal cells in response to poliovirus infection was associated with reduced virus production and better infection control in human patients [53]. On the other hand, knockdown of expression of Atg7 in HeLa cells was associated with a drop of enterovirus D68 replication and non-lytic release [35], underscoring the complex interplay of enteroviruses with the autophagy machinery, and that the viruses may differently benefit from its induction or inhibition in different cell types.

Supporting the possible anti-viral effect of autophagy activation are our observations that LC3 clustering is found only in a fraction of infected cells and that it inversely correlates with the level of viral antigens. Given that infection with poliovirus and other enteroviruses induces a rapid inactivation of cellular transcription and translation, autophagy may be a much more relevant element of protection against enterovirus infection than conventional transcription-based antiviral signaling, at least in certain cell types.

Interestingly, we found that the assembled virions strongly colocalize with p62/SQSTM when the virion signal is only beginning to appear, but such colocalization is lost in cells with a massive accumulation of virions. Together with the data that individually expressed VP0 or the whole capsid protein precursor P1 are also recruited to p62/SQSTM-containing structures, this implies that capsid proteins are sequestered early in infection. Enteroviruses are the only animal (+)RNA viruses without an apparent mechanism of the regulation of the amount of capsid proteins, yet the early accumulation of capsid proteins would result in a premature encapsidation of progeny RNA removing it from the translation/replication pool. Other (+)RNA viruses such as alphaviruses, coronaviruses, noroviruses, hepatitis E virus express capsid proteins from a subgenomic RNA that is synthesized during the late stages of infection [4]. In flaviviruses, which have the same arrangement of the polyprotein with the N-terminal part coding for capsid proteins as in picornaviruses, capsid proteins are targeted to the virion assembly sites distinct from the RNA replication sites, thus they do not interfere with the replication process [54,55]. Interestingly, during infection with at least some cardioviruses, which are also members of the *Picornaviridae* family, the frequency of ribosomal frame-shifting increases along with the accumulation of the viral proteins, which essentially turns the viral genome into the RNA coding for only the capsid proteins [56,57]. The p62/SQSTM-dependent sequestration would provide enteroviruses with the means of downregulating the functional capsid proteins early in infection, when the effective RNA replication is particularly important for the successful establishment of infection.

## Data Availability

Not applicable.
